# Chromosomal and genetic anomalies in fetuses with nuchal translucency between 3.0 and 3.4 mm: A systematic review and meta‐analysis

**DOI:** 10.1111/aogs.70339

**Published:** 2026-08-19

**Authors:** Arianna Carta, Lorenza Della Valle, Giuseppe Rizzo, Ilenia Mappa, Giuseppe Maruotti, Maria Elena Flacco, Lamberto Manzoli, Gennaro Cormio, Asma Khalil, Alberto Galindo, Francesco D'Antonio

**Affiliations:** ^1^ Unit of Obstetrics and Gynaecology, Department of Interdisciplinary Medicine University of Bari ‘Aldo Moro’, Policlinico of Bari Bari Italy; ^2^ Center for Fetal Care and High‐Risk Pregnancy University of Chieti Chieti Italy; ^3^ Department of Maternal and Child Health and Urological Sciences Sapienza University of Rome Rome Italy; ^4^ Gynecology and Obstetrics Unit, Department of Public Health University of Naples Federico II Naples Italy; ^5^ Department of Environmental and Preventive Sciences University of Ferrara Ferrara Italy; ^6^ Department of Medical and Surgical Sciences University of Bologna Bologna Italy; ^7^ Obstetrics and Gynaecology Unit, Department of Biomedical Sciences and Human Oncology University of Bari “Aldo Moro”, Piazza Giulio Cesare 11 Bari Italy; ^8^ Fetal Medicine Unit St. George's University of London London UK; ^9^ Fetal Medicine Unit, Department of Obstetrics and Gynecology, Hospital Universitario 12 de Octubre Complutense University Madrid Spain

**Keywords:** cfDNA, chromosomal anomalies, CNVs, nuchal translucency between 3.0 and 3.4 mm, outcome, prenatal invasive testing

## Abstract

**Introduction:**

To report the prevalence of chromosomal anomalies in fetuses with a nuchal translucency (NT) between 3.0 and 3.4 mm and to assess the incremental yield of invasive prenatal diagnosis over cell‐free DNA (cfDNA) in these fetuses.

**Material and Methods:**

MEDLINE, EMBASE, and The Cochrane Library were searched. Inclusion criteria encompassed fetuses with an NT between 3.0 and 3.4 mm undergoing prenatal invasive testing or postnatal genetic assessment. The observed outcomes included common trisomies such as Trisomy 21, 18, and 13, sex chromosomal anomalies (SCAs), rare autosomal anomalies, copy number variants detected by chromosomal microarray (CMA), and single‐gene disorders. We also reported the rate of anomalies potentially detectable by cfDNA. Finally, we planned to perform a subgroup analysis involving cases with isolated NT between 3.0 and 3.4 mm, including only cases undergoing detailed first‐trimester ultrasound assessment. A random‐effects meta‐analysis of proportions was utilized to analyze the data.

**Results:**

Seventeen studies (7007 fetuses) were included. In fetuses with NT measurements between 3.0 and 3.4 mm, chromosomal anomalies were identified in 13.1% (95% CI 9.3–17.3). Common trisomies included Trisomy 21 at 8.0% (95% CI 5.1–11.5), Trisomy 18 at 1.0% (95% CI 0.6–1.7), and Trisomy 13 at 0.52% (95% CI 0.2–1.0) of all chromosomal anomalies. SCAs occurred in 0.58% (95% CI 0.4–0.8), while rare autosomal trisomies and pathogenic or likely pathogenic copy number variants (CNVs) were found in 0.27% (95% CI 0.1–0.6) and 2.5% (95% CI 1.3–3.9), respectively. Additionally, single‐gene disorders detected via NGS/WES were reported in 5.7% (95% CI 3.3–8) of cases with NT between 3.0 and 3.4 mm. When examining only fetuses with an isolated NT between 3.0 and 3.4 mm, all chromosomal anomalies were identified in 12.4% (95% CI 7.9–17.7).

**Conclusions:**

Fetuses with an NT between 3.0 and 3.4 mm showed a high rate of chromosomal anomalies and CNVs, most of which could potentially be detected through cfDNA.

## Graphical abstract

Fetuses with a nuchal translucency between 3.0 and 3.4 mm showed a high rate of chromosomal anomalies and copy number variants, most of which could potentially be detected through cell‐free DNA.

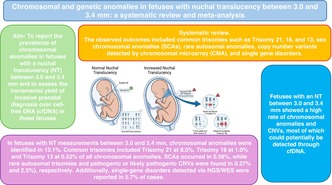

**Abbreviations**
cfDNA: cell‐free DNA
CNVs: copy number variants
NGS: single‐gene disorders detected at next‐generation sequencing
NT: nuchal translucency
RATs: Rare Autosomal Trisomies
SCA: sex chromosomal anomalies
WES: whole exome sequencing

**Key message**

Fetuses with an NT between 3.0 and 3.4 mm had a high prevalence of chromosomal anomalies and CNV. cfDNA can potentially identify the large majority of these anomalies, supporting its role as a second‐line screening test in this population.

## 1 INTRODUCTION

Nuchal translucency (NT) has been a core component of first‐trimester screening for chromosomal anomalies for 40 years.^1^ Increased NT is associated with a higher risk of chromosomal anomalies, mainly Trisomy 21, pathogenic copy number variants (CNVs), and single‐gene disorders, especially those involving the K‐RAS genes.^2^, ^3^, ^4^ Moreover, fetuses with increased NT face a greater likelihood of cardiac and structural anomalies, miscarriage, and fetal death, making this ultrasound parameter a dependable marker of adverse perinatal outcomes.^5^, ^6^

Although multiple screening strategies for fetal aneuploidies exist, the current trend favors measuring NT first, followed by cfDNA testing in patients deemed low risk. In cases where NT is elevated, invasive procedures are recommended.

However, there remains debate on how to define an NT as increased, with some professional societies suggesting using the 95th or 99th percentile, whereas others prefer a more practical cut‐off of 3.0 or 3.5 mm. The International Society of Ultrasound in Obstetrics and Gynecology (ISUOG) guideline on first‐trimester ultrasound advises offering invasive prenatal diagnosis when NT measures 3.5 mm or more.^7^ Conversely, the Society for Maternal‐Fetal Medicine (SMFM) recommends invasive procedures when the NT is 3 mm or greater.^8^

In this context, we conducted a systematic review and meta‐analysis of the published literature reporting the incidence of chromosomal and genetic anomalies in fetuses with NT between 3.0 and 3.4 mm, examining whether the use of cell‐free DNA (cfDNA) in these cases can reliably exclude such anomalies.

## 2 MATERIAL AND METHODS

### 2.1 Protocol, information sources, and literature search

This review was performed according to an a priori designed protocol recommended for systematic reviews and meta‐analyses.^9^, ^10^ MEDLINE, EMBASE, and The Cochrane Library were searched electronically on December 01, 2025, utilizing combinations of the relevant medical subject heading (MeSH) terms, keywords, and word variants for “nuchal translucency,” “chromosomal anomalies,” and “outcome.” The search and selection criteria were restricted to the English language. Reference lists of relevant articles and reviews were searched manually for additional reports. Preferred Reporting Items for Systematic Reviews and Meta‐Analyses (PRISMA) guidelines were followed.^11^ The study was registered with the PROSPERO database (registration number: CRD420261332691).

### 2.2 Outcomes measures

Inclusion criteria were fetuses with an NT between 3.0 and 3.4 mm undergoing prenatal invasive testing.

The outcomes observed were

- Common trisomies, including Trisomy 21, 18, and 13
- Sex chromosomal anomalies (SCA)
- Rare autosomal anomalies
- Copy number variation detected at chromosomal microarray, including microdeletions and microduplications
- Single‐gene disorders detected at next‐generation sequencing (NGS) or whole‐exome sequencing (WES)

Furthermore, we reported the rate of anomalies potentially detectable by cfDNA. For the purpose of this analysis, we base our analysis upon previous large cohort studies, systematic reviews and meta‐analyses on the diagnostic accuracy of cfDNA for common trisomies, SCAs, RATs, and CNV.^12^, ^13^, ^14^, ^15^, ^16^ We decided not compute the diagnostic yield of cfDNA analysis in detecting single‐gene disorders in view of the lack of robust data on the diagnostic accuracy of cfDNA analysis in detecting these disorders.

Finally, we planned to perform a subgroup analysis including only cases with isolated NT between 3.0 and 3.4 mm, defined as those not presenting anomalies potentially detectable at first trimester ultrasound assessment.

### 2.3 Data extraction

Two independent investigators (AC, LDV) examined the abstracts of all potentially relevant articles for suitability. Articles were excluded at this stage only if they did not meet the inclusion criteria. Full‐text copies of the remaining articles were obtained and assessed independently for content, data extraction, and analysis. Disagreements between the two investigators were resolved by discussion with a third author (FDA). Study characteristics were extracted using a predesigned data extraction protocol. If more than one study was published on the same cohort with identical endpoints, only the report containing the most comprehensive information on the population was included to avoid overlapping populations.

We included studies reporting the occurrence of chromosomal or genetic anomalies in fetuses with NT between 3.0 and 3.4 mm. Only full‐text articles were considered eligible for inclusion. Case reports, conference abstracts, and case series with fewer than five cases were also excluded to avoid publication bias. We also excluded studies published before 2015 because we assume that advances in the prenatal imaging techniques and genetic analysis made over that time render older studies less relevant.

### 2.4 Quality assessment

Quality assessment of the included studies was performed using the Newcastle–Ottawa Scale (NOS) for cohort studies.^17^ According to the NOS, each study is judged on three broad domains: selection of the study groups, comparability of the groups, and ascertainment of the outcome of interest. Assessment of the selection domain includes evaluation of the representativeness of the exposed cohort, selection of the nonexposed cohort (in the case of a case–control study), ascertainment of exposure, and demonstration that the outcome of interest was not present at the start of study. Assessment of the comparability domain includes evaluation of the comparability of the cohorts based on the design or analysis. Finally, assessment of the outcome domain includes evaluation of the type of assessment of the outcome of interest and the length and adequacy of follow‐up. According to the NOS, a study can be awarded a maximum of one star for each numbered item within the selection and outcome domains, and a maximum of two stars can be given for comparability.^17^

### 2.5 Statistical analysis

Random‐effects meta‐analysis of proportions was used to combine data and estimate the pooled rates of each anomaly, detected through prenatal invasive testing, along with 95% confidence intervals (CIs). Funnel plots displaying the outcome rate from individual studies vs their precision (1/standard error) were used with an exploratory aim only for the meta‐analyses including ≥10 studies, while tests for funnel plot asymmetry were not used when the total number of publications included for each outcome was fewer than 10. In this case, indeed, the power of such tests is too low to distinguish chance from real asymmetry.^18^, ^19^

Between‐study heterogeneity was explored using the *I* ^2^ statistic, which represents the percentage of between‐study variation that is due to heterogeneity rather than chance. A value of 0% indicates no observed heterogeneity, whereas *I* ^2^ values of ≥50% indicate a substantial level of heterogeneity.^18^, ^19^

As an additional analysis, we used the computed pooled estimates to impute the rates of each chromosomal or genetic anomaly potentially detectable by cfDNA. Accordingly, the diagnostic accuracy of cfDNA to predict common trisomies, SCAs, RATs, and CNVs was extracted from previously published systematic reviews and meta‐analyses. Based on this data, the pooled sensitivity of cfDNA is 99.0% (Trisomy 21), 90% (Trisomy 18), and 94.7% (Trisomy 13), and 99%, 75%, and 77% for SCAs, RATs, and CNVs, respectively. We then applied these estimates to the raw proportion of diseased fetuses reported in each study (and identified by prenatal invasive testing—taken as the gold standard) to recalculate the number of true positive fetuses potentially diagnosed through cfDNA. Finally, these inferred data were used to perform random‐effects meta‐analyses of proportions to obtain the pooled rates (and 95% CIs) of each anomaly if detected with cfDNA. The same approach described above was also used to perform a subgroup analysis, including only those fetuses with isolated NT between 3.0 and 3.4 mm who underwent detailed first‐trimester ultrasound assessment.

Statistical analysis was performed using StatsDirect Statistical Software (StatsDirect Ltd., Cambridge, UK).

## 3 RESULTS

### 3.1 Study selection and characteristics

**FIGURE 1:**
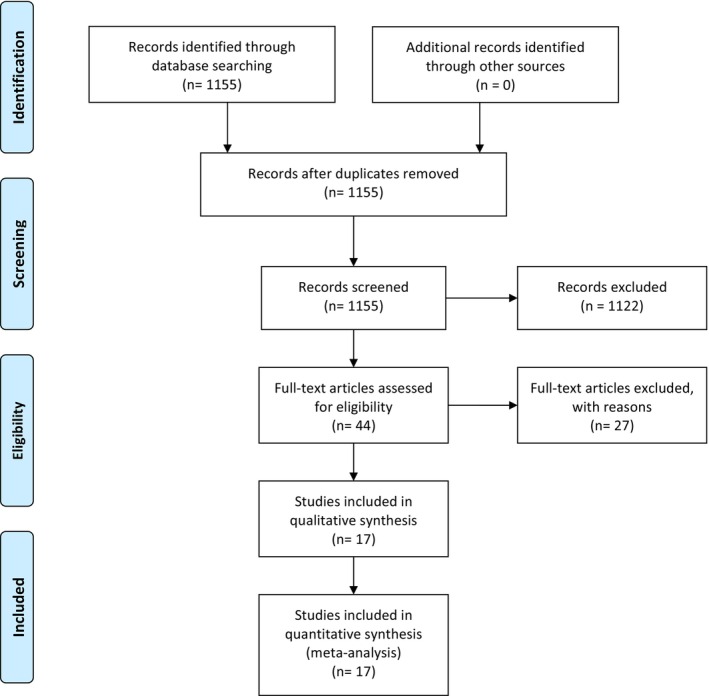
PRISMA flow diagram. Adapted from Ref. [20].

The literature review identified 1155 articles, with 1122 excluded because their outcomes were not relevant, leaving 44 for full‐text review. After further exclusion of 27 articles, 17 studies remained for inclusion in the systematic review (Figure 1, Tables 1 and S1).^21^, ^22^, ^23^, ^24^, ^25^, ^26^, ^27^, ^28^, ^29^, ^30^, ^31^, ^32^, ^33^, ^34^, ^35^, ^36^, ^37^ Removing potential duplicates, a total of 8780 fetuses with NT measurements between 3.0 and 3.4 mm were included, of which outcomes were reported for 7007 fetuses with increased NT regardless of the presence of associated fetal anomalies and for 2429 cases with no associated structural anomalies at the time of the first trimester scan. All studies used standard G‐banded karyotyping or CMA testing, but none reported on single‐gene disorders detected by whole‐exome sequencing (WES) or next‐generation sequencing (NGS).

**TABLE 1:** General characteristics of studies included in systematic review and meta‐analysis.

Author	Year	Country	Study design	Period considered	Structural anomalies	Genetic testing	Fetuses
Jing^21^	2025	China	Retrospective	Not reported	Exluded	G‐banded karyotype, CMA, WES/NGS	604
Vriendt^22^	2025	France	Retrospective	2017–2022	Excluded	G‐banded karyotype, CMA	84
Bellai‐Dussault^23^	2024	Canada	Retrospective	2016–2021	Included	G.banded karyotype, CMA	1789
Huang^24^	2024	China	Retrospective	2014–2022	Excluded	G‐banded karyotype, CMA	470
Rybak‐Krzyszkowska^25^	2024	Czech Republic, Poland, Portugal, Spain	Retrospective	Not reported	Excluded	G‐banded karyotype, CMA	271
Wójtowicz^26^	2024	Poland	Retrospective	2018–2021	Excluded	G‐banded karyotype, CMA	187
Ji^27^	2023	China	Retrospective	2019–2023	Included	G‐banded karyotype, CMA, WES/NGS	716
Xu^28^	2023	China	Retrospective	2016–2021	Included	G.banded karyotype, CMA	107
Hui^29^	2021	Australia	Retrospective	2015–2016	Included	G.banded karyotype, CMA	534
Su^30^	2021	China	Retrospective	2008–2020	Included	G.banded karyotype	274
Sagi‐Dai^31^	2021	Israel	Retrospective	2013–2018	Excluded	CMA	619
Petersen^32^	2020	The Netherlands/Denmark	Retrospective	2012–2019	Excluded	G‐banded karyotype, CMA	211
Grossman^33^	2020	United States	Retrospective	2015–2017	Incldued	G‐banded karyotype, CMA, WES/NGS	31
Zhao^34^	2019	China	Retrospective	2015–2017	Included	G‐banded karyotype, CMA	110
Zhang^35^	2019	China	Retrospective	2015–2018	Included	QF‐PCR, CMA	146
Maya^36^	2017	Israel	Retrospective	2011–2015	Included	G‐banded karyotype, CMA	170
Berger^37^	2016	United States	Retrospective	2010–2014	Included	G‐banded karyotype,CMA	2644

*Note*: Only first author is given for each study.

Abbreviations: NGS, next‐generation sequencing; WES, whole‐exome sequencing.

The results of the quality assessment of the included studies using the NOS are shown in Table 2. The studies demonstrated an overall good score regarding the selection of study groups and the determination of the outcome of interest. The main limitations of most included studies were their retrospective design, small sample sizes, and lack of correlation with the occurrence of structural anomalies either at first or second trimester ultrasound. Test for publication bias are reported in Supplementary material 2.

**TABLE 2:** Methodological assessment of included studies based on Newcastle–Ottawa Score (NOS).

Author	Year	Selection	Comparability	Outcome
Jing^21^	2025	★★★	★★	★★★
Vriendt^22^	2025	★★	★	★★
Bellai‐Dussault^23^	2024	★★	★★	★★
Huang^24^	2024	★★	★	★★
Rybak‐Krzyszkowska^25^	2024	★★	★★	★★
Wójtowicz^26^	2024	★★	★	★★
Ji^27^	2023	★★	★	★★
Xu^28^	2023	★★	★	★★
Hui^29^	2021	★★	★	★
Su^30^	2021	★★	★★	★★
Sagi‐Dai^31^	2021	★★	★	★
Petersen^32^	2020	★★	★	★★
Grossman^33^	2020	★★	★	★★
Zhao^34^	2019	★★	★	★★
Zhang^35^	2019	★★	★	★
Maya^36^	2017	★★	★★	★★
Berger^37^	2016	★★	★	★

### 3.2 Synthesis of results

**FIGURE 2:**
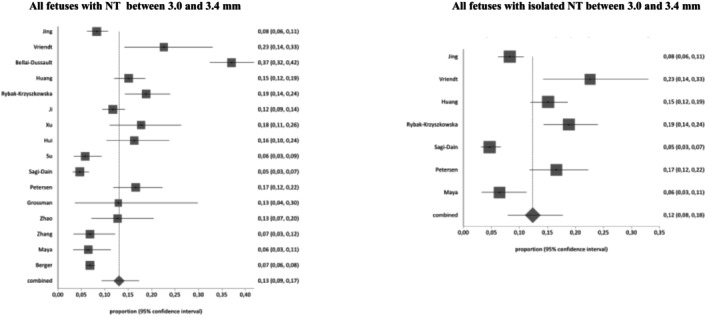
Pooled proportions for the occurrence of all chromosomal anomalies in all fetuses with nuchal translucency between 3.0 and 3.4 mm and in those not presenting with associated structural anomalies at the first trimester scan. Adapted from Ref. [20].

Across all fetuses with NT measurements between 3.0 and 3.4 mm, chromosomal anomalies were found in 13.1% (95% CI 9.3–17.3) (Figure 2). Common trisomies were present in 10.1% (95% CI 6.6–14.2), with Trisomy 21 accounting for 8.0% (95% CI 5.1–11.5), Trisomy 18 for 1.0% (95% CI 0.6–1.7), and Trisomy 13 for 0.52% (95% CI 0.2–1.0) of all chromosomal anomalies. SCAs were reported in 0.58%% (95% CI 0.4–0.8), while the corresponding figures for rare autosomal trisomies and pathogenic or likely pathogenic CNVs were 0.27% (95% CI 0.1–0.6) and 2.5% (95% CI 1.3–3.9) of cases (Table 3). Finally, single‐gene disorders at NGS/WES were reported in 5.7% (95% CI 3.3–8.9) of cases with NT between 3.0 and 3.4 mm.

**TABLE 3:** Pooled proportions for the occurrence of different chromosomal anomalies in fetuses with a nuchal translucency between 3.0 and 3.4 mm.

Anomaly	Detected by genetic testing				Potentially identifiable by cfDNA			Incremental yield of invasive prenatal diagnosis (%)
	Studies^(ref)^	Fetuses (n/N)	Pooled proportion (95% CI)	*I* ^2^ (%)	Fetuses (n/N)	(95% CI)	*I* ^2^ (%)	
**All fetuses with a nuchal translucency between 3.0 and 3.4 mm**								
All anomalies	16^21^, ^22^, ^23^, ^24^, ^25^, ^27^, ^28^, ^29^, ^30^, ^31^, ^32^, ^33^, ^34^, ^35^, ^36^, ^37^	771/7007	13.06 (9.31–17.33)	95.3	–	–	–	–
Common trisomies	15^21^, ^22^, ^23^, ^24^, ^25^, ^28^, ^29^, ^30^, ^31^, ^32^, ^33^, ^34^, ^35^, ^36^, ^37^	547/6291	10.06 (6.61–14.15)	95.2	–	–	–	–
Trisomy 21	14^21^, ^22^, ^23^, ^24^, ^25^, ^28^, ^29^, ^31^, ^32^, ^33^, ^34^, ^35^, ^36^, ^37^	449/6162	8.02 (5.10–11.53)	94.4	449/6162	8.02 (5.10–11.53)	94.4	0
Trisomy 18	14^21^, ^22^, ^23^, ^24^, ^25^, ^28^, ^29^, ^31^, ^32^, ^33^, ^34^, ^35^, ^36^, ^37^	51/6162	1.04 (0.56–1.67)	69.8	51/6162	1.04 (0.56–1.67)	69.8	0
Trisomy 13	14^21^, ^22^, ^23^, ^24^, ^25^, ^28^, ^29^, ^31^, ^32^, ^33^, ^34^, ^35^, ^36^, ^37^	28/6162	0.52 (0.21–0.97)	67.8	28/6162	0.52 (0.21–0.97)	67.8	0
Sex chromosomal anomalies	15^21^, ^22^, ^23^, ^24^, ^25^, ^28^, ^29^, ^30^, ^31^, ^32^, ^33^, ^34^, ^35^, ^36^, ^37^	31/6291	0.58 (0.39–0.81)	8.2	31/6291	0.58 (0.39–0.81)	8.2	0
Rare autosomal trisomies	13^21^, ^22^, ^24^, ^25^, ^28^, ^30^, ^31^, ^32^, ^33^, ^34^, ^35^, ^36^, ^37^	10/5596	0.27 (0.08–0.57)	50.9	8/5596	0.23 (0.07–0.48)	50.9	0.04
Pathogenic or likely pathogenic CNV	15^21^, ^22^, ^23^, ^24^, ^25^, ^27^, ^28^, ^30^, ^31^, ^32^, ^33^, ^34^, ^35^, ^36^, ^37^	121/6733	2.47 (1.34–3.92)	89.3	94/6733	1.99 (1.10–3.15)	86.2	0.48
Single‐gene disorders	2^21^, ^27^	14/259	5.74 (3.25–8.89)	0	–	–	–	–
**Fetuses with apparently isolated nuchal translucency between 3.0 and 3.4 mm**								
All anomalies	7^21^, ^22^, ^24^, ^25^, ^31^, ^32^, ^36^	266/2429	12.38 (7.89–17.72)	92.3	–	–	–	–
Common trisomies	7^21^, ^22^, ^24^, ^25^, ^31^, ^32^, ^36^	165/2429	7.91 (4.54–12.12)	91.3	–	–	–	–
Trisomy 21	7^21^, ^22^, ^24^, ^25^, ^31^, ^32^, ^36^	140/2429	6.57 (3.73–10.22)	89.9	140/2429	6.57 (3.73–10.22)	89.9	0
Trisomy 18	7^21^, ^22^, ^24^, ^25^, ^31^, ^32^, ^36^	18/2429	0.82 (0.26–1.69)	71.9	18/2429	0.82 (0.26–1.69)	71.9	0
Trisomy 13	7^21^, ^22^, ^24^, ^25^, ^31^, ^32^, ^36^	7/2429	0.31 (0.05–0.78)	57.9	7/2429	0.31 (0.05–0.78)	57.9	0
Sex chromosomal anomalies	7^21^, ^22^, ^24^, ^25^, ^31^, ^32^, ^36^	15/2429	0.73 (0.43–1.10)	0	15/2429	0.73 (0.43–1.10)	0	0
Rare autosomal trisomies	7^21^, ^22^, ^24^, ^25^, ^31^, ^32^, ^36^	9/2429	0.39 (0.16–0.71)	15	7/2429	0.34 (0.15–0.61)	0	0.05
Pathogenic or likely pathogenic CNV	7^21^, ^22^, ^24^, ^25^, ^31^, ^32^, ^36^	78/2429	3.39 (2.17–4.85)	67.4	60/2429	2.60 (1.70–3.69)	54.3	0.79
Single‐gene disorders	1^21^	–	–	–	–	–		–

When examining only fetuses with an isolated NT between 3.0 and 3.4 mm, all chromosomal anomalies were identified in 12.4% (95% CI 7.9–17.7), with common trisomies occurring in 7.9% (95% CI 4.5–12.1) of all cases. Trisomy 21 was detected in 6.6% (95% CI 3.7–10.2), Trisomy 18 in 0.82% (95% CI 0.3–1.7), and Trisomy 13 in 0.31% (95% CI 0.1–0.8) (Table 3).

SCAs were reported in 0.73% (95% CI 0.4–1.1), while the corresponding figures for rare autosomal trisomies and pathogenic or likely pathogenic CNVs were 0.39% (95% CI 0.2–0.7) and 3.4% (95% CI 2.2–4.9). Unfortunately, we could not compute a pooled data synthesis of the rate of single‐gene disorders at NGS/WES analysis as only one study reported this information.^21^

The rate of anomalies potentially detectable by cfDNA, as reported in the published literature, is shown in Table 3.

When examining all fetuses with an NT between 3.0 and 3.4 mm, the additional yield of invasive prenatal diagnosis over cfDNA was 0% for Trisomy 21, Trisomy 18, Trisomy 13, and SCAs, 0.04% for RATs, and 0.48% for pathogenic or likely pathogenic CNVs. Finally, when including only fetuses with an isolated NT within this range, the additional yield of invasive prenatal diagnosis over cfDNA remained 0% for Trisomy 21, Trisomy 18, Trisomy 13, and SCAs, 0.03% for RATs, and 0.84% for pathogenic or likely pathogenic CNVs.

## 4 DISCUSSION

The findings from this systematic review showed that fetuses with an NT between 3.0 and 3.4 mm have a relatively high prevalence, about 10–12% of chromosomal anomalies. Common trisomies, especially Trisomy 21, account for the vast majority (5%) of these anomalies, while the rates of SCAs, RATS, and CNVs are 0.7%, 0.4%, and 2.6%. cfDNA can potentially identify most of these anomalies, with only a 0.8% decrease in detection rate for CNVs, thus supporting its role in screening for chromosomal anomalies even in these fetuses.

However, the limited data on single‐gene disorders may restrict the clinical relevance of these findings, needing validation in larger studies.

This is, to the best of our knowledge, the first systematic review reporting the rate of chromosomal anomalies in fetuses presenting with an NT between 3.0 and 3.4 mm.

The main strengths of the present systematic review are the multitude of chromosomal anomalies explored and the stratification of the analysis according to the presence of structural anomalies at first trimester ultrasound and thorough literature search, while its weaknesses are the small number of cases in some of the included studies, their retrospective nonrandomized design, and lack of stratification of the analysis according to the week at ultrasound assessment, and presence and type of associated structural anomalies, at least for most of the included studies. Notably, most of the included studies did not specifically report the imaging protocol adopted in the first trimester, and it may be entirely possible that some of the cases labeled as isolated had structural anomalies. Likewise, the lack of information on second‐trimester ultrasound did not allow for a meaningful subgroup analysis considering only cases with negative ultrasound both in the first and second trimester. Another major limitation of the present systematic review is that the occurrence of genetic anomalies in the population included in our analysis may not fully reflect the actual prevalence of such anomalies in all fetuses presenting with an NT between 3.0 and 3.4 mm, as not all these fetuses undergo genetic testing, being traditionally considered not at very high risk of chromosomal or genetic anomalies. The assessment of chromosomal or genetic anomalies was also limited because not all these disorders are detected at birth, and postnatal testing was not conducted on the entire study population. The analysis of single‐gene disorders in all fetuses with NT between 3.0 and 3.4 mm was limited by the very small number of cases included. It is also possible that mainly cases showing anomalies later in the second trimester or with a higher risk of genetic anomalies were referred for testing, which could potentially lead to an overestimation of the occurrence of such anomalies in this subset of fetuses. Unfortunately, we were unable to perform any pooled data analysis on the association with single‐gene disorders in fetuses with an isolated NT of 3.0–3.4 mm, as this information was not reported in any of the included studies. Finally, we could only impute the rate of anomalies potentially detected through cfDNA and, although this procedure has shown an excellent diagnostic accuracy as a whole, further research, specifically focused on fetuses with NT between 3.0 and 4.0, is certainly needed to confirm its supporting role also for this subset of chromosomal screening procedures.

NT has served as a proxy for risk stratification of chromosomal, genetic, and structural anomalies for decades. Fetuses with increased NT are at higher risk of being affected by such anomalies compared to those with NT measurements within the normal range. In this context, invasive prenatal diagnosis is often offered to parents to exclude the presence of chromosomal or genetic anomalies. Genetic testing in these fetuses involves not only standard karyotyping and CMA but also the assessment for single‐gene disorders, primarily those involving K‐RAS genes, given the association between increased NT and these anomalies. Furthermore, international guidelines recommend that early anomaly scans and fetal echocardiography should be offered to these parents because of the increased risk of structural anomalies, mainly congenital heart defects, in fetuses with increased NT.

However, whereas such assessment is commonly performed in fetuses with NT ≥3.5 mm, there is still significant clinical variability in the management of fetuses with NT at the upper range of normality below 3.5 mm. International guidelines differ regarding the cut‐offs used to define an NT as increased and the management of these fetuses.

ISUOG guidelines on first‐trimester ultrasound recommend offering invasive testing only when the NT is ≥3.5 mm, whereas the SMFM and ACOG suggest offering invasive genetic testing when the NT is ≥3.0 mm.^7^, ^8^, ^38^

In the present systematic review, chromosomal anomalies occurred in 12% of fetuses with an NT between 3.0 and 3.4 mm, mostly represented by common trisomies, especially Trisomy 21, while the risk of pathogenic or likely pathogenic CNVs was about 3%. In this scenario, the application of cfDNA could detect all common trisomies, but it would not identify less than 1% of CNVs, assuming cfDNA has a sensitivity of about 77% for these anomalies. However, the lack of data on second‐trimester ultrasound assessment for most of the included studies did not allow us to elucidate whether cases affected by CNVs showed associated anomalies at the scan.

The association between increased NT and single‐gene disorders presents another important issue. Previous meta‐analyses have reported an incidence of 4–8% of anomalies, mainly related to K‐RAS genes, detected solely through NGS/WES in fetuses with increased NT and normal CMA, with the risk rising alongside the severity of NT thickness.^3^, ^4^ This highlights the need to offer invasive prenatal diagnosis, as there is still no solid data on the actual diagnostic accuracy of cfDNA in detecting these anomalies. Importantly, fetuses with K‐RAS gene anomalies showed subtle or no specific anomalies at the scan, with the most common sign being increased NT.^39^ In the present systematic review, the risk of single‐gene disorders in all fetuses with NT between 3.0 and 3.4, regardless of the presence of anomalies, was about 5%, whereas it was not possible to perform a pooled‐data synthesis for fetuses with isolated NT.

Based on these findings, a feasible clinical strategy for first trimester screening could involve offering NT and cfDNA testing for common trisomies to couples whose fetuses have an NT less than 3.0 mm. For fetuses with NT measurements between 3.0 and 3.4 mm, cfDNA with CNV analysis might be offered as an alternative to invasive testing, provided structural anomalies are ruled out. It is important to discuss with parents the limitations of cfDNA, especially the possibility of missed chromosomal abnormalities such as certain CNVs and single‐gene disorders. Conversely, invasive prenatal diagnosis should be recommended for cases presenting structural anomalies.

## 5 CONCLUSION

Fetuses with an NT between 3.0 and 3.4 mm have a relatively high prevalence of chromosomal anomalies, which can potentially be identified by cfDNA, whereas there is limited evidence for an association with single‐gene disorders. The findings from this systematic review suggest that offering cfDNA testing for common trisomies and CNVs may be a reasonable option for fetuses with an NT between 3.0 and 3.4 mm after exclusion of structural anomalies, although this requires validation in large population studies.

## AUTHOR CONTRIBUTIONS

Arianna Carta: conceived the present idea, investigated, performed the analysis, drafted and revised the manuscript. Lorenza Della Valle: investigator, performed the analysis, discussed the results, revised the manuscript. Giuseppe Rizzo: supervised the findings of this work, revised the manuscript, and discussed the results. Ilenia Mappa: supervised the findings of this work, revised the manuscript. Giuseppe Maruotti: supervised the findings of this work, revised the manuscript. Maria Elena Flacco: performed the analysis, supervised the findings of this work, revised the manuscript. Lamberto Manzoli: supervised the findings of this work, revised the manuscript. Gennaro Cormio: supervised the findings of this work, revised the manuscript. Asma Khalil: supervised the findings of this work, revised the manuscript, discussed the results. Alberto Galindo: supervised the findings of this work, revised the manuscript. Francesco D'Antonio: conceived the present idea, performed the analysis, supervised the findings of this work, drafted and revised the manuscript.

## FUNDING INFORMATION

No funding was received for this work.

## CONFLICT OF INTEREST STATEMENT

No conflict of interest to declare by any of the authors.

## Supporting information


**Data S1:** 5_Supplementary material_funnel_plots.

**Supplementary Table 1.** Excluded studies and the reason for the exclusion.

## Data Availability

The data that support the findings of this study are available from the corresponding author upon reasonable request.

## References

[1] Nicolaides KH , Snijders RJ , Gosden CM , Berry C , Campbell S . Ultrasonographically detectable markers of fetal chromosomal abnormalities. Lancet. 1992;340(8821):704‐707. 1355807 10.1016/0140-6736(92)92240-g

[2] Grande M , Jansen FA , Blumenfeld YJ , et al. Genomic microarray in fetuses with increased nuchal translucency and normal karyotype: a systematic review and meta‐analysis. Ultrasound Obstet Gynecol. 2015;46(6):650‐658. 25900824 10.1002/uog.14880

[3] Di Girolamo R , Rizzo G , Khalil A , et al. Whole exome sequencing in fetuses with isolated increased nuchal translucency: a systematic review and meta‐analysis. J Matern Fetal Neonatal Med. 2023;36(1):2193285. 37019452 10.1080/14767058.2023.2193285

[4] Pauta M , Martinez‐Portilla RJ , Borrell A . Diagnostic yield of next‐generation sequencing in fetuses with isolated increased nuchal translucency: systematic review and meta‐analysis. Ultrasound Obstet Gynecol. 2022;59(1):26‐32. 34309942 10.1002/uog.23746

[5] Sotiriadis A , Papatheodorou S , Eleftheriades M , Makrydimas G . Nuchal translucency and major congenital heart defects in fetuses with normal karyotype: a meta‐analysis. Ultrasound Obstet Gynecol. 2013;42(4):383‐389. 23606595 10.1002/uog.12488

[6] Matarrelli B , Khalil A , Bernassola M , et al. Outcome of fetuses with early increased nuchal translucency: systematic review and meta‐analysis. Ultrasound Obstet Gynecol. 2025;66(2):147‐154. 40519157 10.1002/uog.29260

[7] International Society of Ultrasound in Obstetrics and Gynecology , Bilardo CM , Chaoui R , et al. ISUOG Practice Guidelines (updated): performance of 11‐14‐week ultrasound scan. Ultrasound Obstet Gynecol. 2023;61(1):127‐143. 36594739 10.1002/uog.26106

[8] Rink BD , Dugoff L , Kuller JA , Society for Maternal‐Fetal Medicine (SMFM) Publications Committee . Society for Maternal‐Fetal Medicine Consult Series #74: cell‐free DNA screening for aneuploidies: updated guidance. Preganncy (Hoboken). 2025;1(6):e70139. 10.1002/pmf2.70139 PMC13344640 42597105

[9] Henderson LK , Craig JC , Willis NS , Tovey D , Webster AC . How to write a cochranesystematic review. Nephrology (Carlton). 2010;15:617‐624. 20883282 10.1111/j.1440-1797.2010.01380.x

[10] NHS Centre for Reviews and Dissemination . Systematic Reviews: CRD's Guidance for Undertaking Reviews in Health Care. University of York; 2009. https://www.york.ac.uk/media/crd/Systematic_Reviews.pdf

[11] Page MJ , McKenzie JE , Bossuyt PM , et al. Updating guidance for reporting systematic reviews: development of the PRISMA 2020 statement. J Clin Epidemiol. 2021;134:103‐112. 33577987 10.1016/j.jclinepi.2021.02.003

[12] Gil MM , Accurti V , Santacruz B , Plana MN , Nicolaides KH . Analysis of cell‐free DNA in maternal blood in screening for aneuploidies: updated meta‐analysis. Ultrasound Obstet Gynecol. 2017;50(3):302‐314. 28397325 10.1002/uog.17484

[13] Shear MA , Swanson K , Garg R , et al. A systematic review and meta‐analysis of cell‐free DNA testing for detection of fetal sex chromosome aneuploidy. Prenat Diagn. 2023;43(2):133‐143. 36588186 10.1002/pd.6298 PMC10268789

[14] Raymond YC , Fernando S , Menezes M , et al. Cell‐free DNA screening for rare autosomal trisomies and segmental chromosome imbalances. Prenat Diagn. 2022;42(11):1349‐1357. 36068932 10.1002/pd.6233 PMC9826090

[15] Della Valle L , Piergianni M , Khalil A , et al. Accuracy of cell‐free fetal DNA in detecting chromosomal anomalies in women experiencing miscarriage: systematic review and meta‐analysis. Ultrasound Obstet Gynecol. 2025;65(1):13‐19. 39644510 10.1002/uog.29131

[16] Raymond YC , Acreman ML , Bussolaro S , et al. The accuracy of cell‐free DNA screening for fetal segmental copy number variants: a systematic review and meta‐analysis. BJOG. 2023;130(6):549‐559. 36655363 10.1111/1471-0528.17386

[17] Newcastle‐Ottawa Scale for assessing the quality of nonrandomised studies in meta‐ analyses. http://www.ohri.ca/programs/clinical_epidemiology/oxford.asp 10.1007/s10654-010-9491-z 20652370

[18] Manzoli L , De Vito C , Salanti G , D'Addario M , Villari P , Ioannidis JP . Meta‐analysis of the immunogenicity and tolerability of pandemic influenza a 2009 (H1N1) vaccines. PLoS One. 2011;6:e24384. 21915319 10.1371/journal.pone.0024384 PMC3167852

[19] Hunter JP , Saratzis A , Sutton AJ , Boucher RH , Sayers RD , Bown MJ . In meta‐analyses of proportion studies, funnel plots were found to be an inaccurate method of assessing publication bias. J Clin Epidemiol. 2014;67:897‐903. 24794697 10.1016/j.jclinepi.2014.03.003

[20] Moher D , Liberati A , Tetzlaff J , Altman DG , The PRISMA Group . Preferred Reporting Items for Systematic Reviews and Meta‐Analyses: The PRISMA Statement. PLoS Med. 2009;6(6):e1000097. doi:10.1371/journal.pmed1000097 21603045 PMC3090117

[21] Jing XY , Xiao ZQ , Li DZ . Invasive genetic testing for isolated increased nuchal translucency of 3.0‐3.4 mm: results from cohort analysis with 604 fetuses. Int J Gynaecol Obstet. 2025;168(3):1331‐1333. 39295216 10.1002/ijgo.15928

[22] Vriendt MDE , Rooryck C , Madar H , et al. Outcomes associated with fetal nuchal translucency between 3.0 and 3.4 mm in the first trimester. Acta Obstet Gynecol Scand. 2025;104(4):629‐636. 39962853 10.1111/aogs.15055 PMC11919737

[23] Bellai‐Dussault K , Dougan SD , Fell DB , et al. Ultrasonographic fetal nuchal translucency measurements and cytogenetic outcomes. JAMA Netw Open. 2024;7(3):e243689. 38530313 10.1001/jamanetworkopen.2024.3689 PMC10966411

[24] Huang J , Wu D , He JH , et al. Associations between genomic aberrations, increased nuchal translucency, and pregnancy outcomes: a comprehensive analysis of 2,272 singleton pregnancies in women under 35. Front Med (Lausanne). 2024;11:1376319. 38633307 10.3389/fmed.2024.1376319 PMC11021699

[25] Rybak‐Krzyszkowska M , Madetko‐Talowska A , Szewczyk K , et al. Is nuchal translucency of 3.0‐3.4 mm an indication for cfDNA testing or microarray?—a multicenter retrospective clinical cohort study. Fetal Diagn Ther. 2024;51(5):453‐462. 38815555 10.1159/000539463 PMC11446333

[26] Wójtowicz A , Kowalczyk K , Szewczyk K , et al. Array comparative genomic hybridization (aCGH) results among patients referred to invasive prenatal testing after first‐trimester screening: a comprehensive cohort study. Diagnostics (Basel). 2024;14(19):2186. 39410589 10.3390/diagnostics14192186 PMC11475562

[27] Ji X , Li Q , Qi Y , et al. When NIPT meets WES, prenatal diagnosticians face the dilemma: genetic etiological analysis of 2,328 cases of NT thickening and follow‐up of pregnancy outcomes. Front Genet. 2023;14:1227724. 37600658 10.3389/fgene.2023.1227724 PMC10433188

[28] Xu Y , Hu S , Chen L , et al. Application of non‐invasive prenatal testing in screening chromosomal aberrations in pregnancies with different nuchal translucency cutoffs. Mol Cytogenet. 2023;16(1):29. 37898768 10.1186/s13039-023-00661-1 PMC10613380

[29] Hui L , Pynaker C , Bonacquisto L , et al. Reexamining the optimal nuchal translucency cutoff for diagnostic testing in the cell‐free DNA and microarray era: results from the Victorian Perinatal Record Linkage study. Am J Obstet Gynecol. 2021;225(5):527.e1‐527.e12. 10.1016/j.ajog.2021.03.050 33957116

[30] Su L , Wu X , Lin N , et al. Different cutoff values for increased nuchal translucency in first‐trimester screening to predict fetal chromosomal abnormalities. Int J Gen Med. 2021;14:8437‐8443. 34819751 10.2147/IJGM.S330960 PMC8608408

[31] Sagi‐Dain L , Singer A , Ben Shachar S , et al. Risk of clinically significant chromosomal microarray analysis findings in fetuses with nuchal translucency from 3.0 mm through 3.4 mm. Obstet Gynecol. 2021;137(1):126‐131. 33278279 10.1097/AOG.0000000000004195

[32] Petersen OB , Smith E , Van Opstal D , et al. Nuchal translucency of 3.0‐3.4 mm an indication for NIPT or microarray? Cohort analysis and literature review. Acta Obstet Gynecol Scand. 2020;99(6):765‐774. 32306377 10.1111/aogs.13877 PMC7318216

[33] Grossman TB , Bodenlos KL , Chasen ST . Abnormal nuchal translucency: residual risk with normal cell‐free DNA screening. J Matern Fetal Neonatal Med. 2020;33(18):3062‐3067. 30669906 10.1080/14767058.2019.1568405

[34] Zhao XR , Gao L , Wu Y , Wang YL . Application of chromosomal microarray in fetuses with increased nuchal translucency. J Matern Fetal Neonatal Med. 2020;33(10):1749‐1754. 30688128 10.1080/14767058.2019.1569622

[35] Zhang Z , Hu T , Wang J , Li Q , Wang H , Liu S . Prenatal diagnostic value of chromosomal microarray in fetuses with nuchal translucency greater than 2.5 mm. Biomed Res Int. 2019;2019:6504159. 32908864 10.1155/2019/6504159 PMC7471829

[36] Maya I , Yacobson S , Kahana S , et al. Cut‐off value of nuchal translucency as indication for chromosomal microarray analysis. Ultrasound Obstet Gynecol. 2017;50(3):332‐335. 28133835 10.1002/uog.17421

[37] Berger VK , Norton ME , Sparks TN , Flessel M , Baer RJ , Currier RJ . The utility of nuchal translucency ultrasound in identifying rare chromosomal abnormalities not detectable by cell‐free DNA screening. Prenat Diagn. 2020;40(2):185‐190. 31652356 10.1002/pd.5583 PMC7002253

[38] American College of Obstetricians and Gynecologists' Committee on Practice Bulletins—Obstetrics , Committee on Genetics , Society for Maternal‐Fetal Medicine . Screening for fetal chromosomal abnormalities: ACOG practice bulletin, number 226. Obstet Gynecol. 2020;136(4):e48‐e69. 32804883 10.1097/AOG.0000000000004084

[39] Tangshewinsirikul C , Wattanasirichaigoon D , Tim‐Aroon T , et al. Prenatal sonographic features of Noonan syndrome: case series and literature review. J Clin Med. 2024;13(19):5735. 39407794 10.3390/jcm13195735 PMC11476750

