# Supplementary material for: Chromosomal and genetic anomalies in fetuses with nuchal translucency between 3.0 and 3.4 mm: A systematic review and meta‐analysis
Source: Acta Obstet Gynecol Scand. 2026 Aug 19;105(11):2148–57. doi: 10.1111/aogs.70339 (PMC13487402; doi:10.1111/aogs.70339)
Supplement: Supplementary file 1 [file AOGS-105-2148-s001.docx]

**Supplementary Table 1.** Excluded studies and the reason for the exclusion.

| **Author** | **Year** | **Title** | **Reason for the exclusion** |
| --- | --- | --- | --- |
| Xie | 2022 | Application of expanded noninvasive prenatal test in prenatal diagnosis of fetuses with increased nuchal translcuency | This study adopts different cut-offs for reporting chromosomal anomalies (2.5-3.4 mm) |
| Coello‑Cahuao | 2022 | Array study in fetuses with nuchal translucency above the 95th percentile: a 4‑year observational single‑centre study | This study adopts a different cut-off (95th percentile-3.4 mm) compared to that adopted in the present systematic review |
| Zhou | 2022 | Prenatal Genetic Diagnosis of Fetal Cystic Hygroma: A Retrospective Single-Center Study from China | No data on the outcomes observed in the present systematic review could be extrapolated from this study |
| Hai Long | 2021 | Relation Between Increased Fetal Nuchal Translucency Thickness and Chromosomal Defects in Northern Vietnam | This study adopts a different cut-off for reporting chromosomal anomalies (2.5-3.4 mm) |
| Miranda | 2020 | Should cell-free DNA testing be used in pregnancy with increased fetal nuchal translucency? | It was not possible to extrapolate the occurrence of chromosomal anomalies in fetuses with NT between 3.0 and 3.4 mm |
| Bardi | 2020 | Is there still a role for nuchal translucency measurement in the changing paradigm of first trimester screening? | This study adopts a different cut-off (95th percentile) for reporting chromosomal anomalies (2.5-3.4 mm) |
| Lin | 2020 | Detection of copy number variants with chromosomal microarray in 10 377 pregnancies at a single laboratory | No data on the outcomes observed in the present systematic review could be extrapolated from this study |
| Su | 2019 | Clinical application of chromosomal microarray analysis in fetuses with increased nuchal translucency and normal karyotype | This study adopts a different cut-off (95th percentile) for reporting chromosomal anomalies (2.5-3.4 mm) |
| Gadsbøll | 2019 | Current use of noninvasive prenatal testing in Europe, Australia and the USA: A graphical presentation | No data on the outcomes observed in the present systematic review could be extrapolated from this study |
| Stuurman | 2019 | Prenatal ultrasound findings of rasopathies in a cohort of 424 fetuses: update on genetic testing in the NGS era | No data on the outcomes observed in the present systematic review could be extrapolated from this study |
| Chong | 2019 | Prenatal chromosomal microarray testing of fetuses with ultrasound structural anomalies: A prospective cohort study of over 1000 consecutive cases | This study adopts a different cut-off (3.5 mm) for reporting chromosomal anomalies |
| Huang | 2018 | What would be missed in the first trimester if nuchal translucency measurement is replaced by cell free DNA foetal aneuploidy screening? | It was not possible to extrapolate the number of fetuses with NT between 3.0 and 3.4 mm |
| Vogel | 2018 | Chromosomal microarray as primary diagnostic genomic tool for pregnancies at increased risk within a populationbased combined ﬁrst-trimester screening program | No data on the outcomes observed in the present systematic review could be extrapolated from this study |
| Miltoft | 2018 | Contingent first-trimester screening for aneuploidies with cell-free DNA in a Danish clinical setting | No data on the outcomes observed in the present systematic review could be extrapolated from this study |
| Kenkhuis | 2018 | Yield of a 12-13 week scan for the early diagnosis of fetal congenital anomalies in the cell-free DNA era | No data on the outcomes observed in the present systematic review could be extrapolated from this study |
| Sotiriadis | 2017 | Non-invasive prenatal screening versus prenatal diagnosis by array comparative genomic hybridization: a comparative retrospective study | No data on the outcomes observed in the present systematic review could be extrapolated from this study |
| Srebniak | 2016 | Enlarged NT (3,5 ) | Only cases with NT >3.5 mm were included in this study |
| Oepkes | 2016 | Trial by Dutch laboratories for evaluation of non‐invasive prenatal testing Part I | It was not possible to extrapolate the occurrence of genetic anomalies in fetuses with NT between 3.0 and 3.4 mm |
| van Schendel | 2016 | Trial by Dutch laboratories for evaluation of non‐invasive prenatal testing Part II | It was not possible to extrapolate the occurrence of genetic anomalies in fetuses with NT between 3.0 and 3.4 mm |
| Sinajon | 2016 | Increased nuchal translucency: results from microarray and RASopathy disorders testing | Only cases with NT >3.5 mm were included in this study |
| Yang | 2016 | Submicroscopic chromosomal abnormalities in Fetuses with Increased Nuchal Translucency and Normal Karyotype | This study adopts a different cut-off (NT between 3.0 and 3.5 mm) for reporting chromosomal anomalies (2.5-3.4 mm) |
| Bornstein | 2016 | Universal Prenatal Chromosomal Microarray Analysis: Additive Value and Clinical Dilemmas in Fetuses with a Normal Karyotype | No data on the outcomes observed in the present systematic review could be extrapolated from this study |
| Van Opstal | 2015 | False Negative NIPT Results: Risk Figures for Chromosomes 13, 18 and 21 Based on Chorionic Villi Results in 5967 Cases and Literature Review | No data on the outcomes observed in the present systematic review could be extrapolated from this study |
| Lichtenbelt | 2015 | Detection of fetal chromosomal anomalies: does nuchal translucency measurement have added value in the era of non-invasive prenatal testing? | This study adopts a different cut-off (3.5 mm) for reporting chromosomal anomalies |
| Petersen | 2014 | Potential diagnostic consequences of applying non-invasive prenatal testing: population-based study from a country with existing ﬁrst-trimester screening | No data on the outcomes observed in the present systematic review could be extrapolated from this study |
| Yatsenko | 2012 | Application of chromosomal microarray in the evaluation of abnormal prenatal f indings | Th threshold for defining an increased NT was not clearly defined |
| Scand | 2011 | Testing for 22q11 microdeletion in 146 fetuses with nuchal translucency above | Only cases with NT >3.5 mm were included in this study |
