# Supplementary material for: Chromosomal and genetic anomalies in fetuses with nuchal translucency between 3.0 and 3.4 mm: A systematic review and meta‐analysis
Source: Acta Obstet Gynecol Scand. 2026 Aug 19;105(11):2148–57. doi: 10.1111/aogs.70339 (PMC13487402; doi:10.1111/aogs.70339)
Supplement: Supplementary file 2 [file AOGS-105-2148-s002.docx]

**Supplementary material: test for pubblication bias**

***Al fetuses with NT between 3.0 and 3.4 mm***

***All anomalies***

Bias indicators

Begg-Mazumdar: Kendall's 0,333333 P = 0,0788

Egger: bias = 3,887548 (95% CI = 0,927667 to 6,84743) P = 0,0137

Harbord: bias = 3,946265 (92,5% CI = -1,154616 to 9,047147) P = 0,1589

***Common trisomies***

Bias indicators

Begg-Mazumdar: Kendall's 0,371429 P = 0,059

Egger: bias = 3,340733 (95% CI = 0,249009 to 6,432456) P = 0,0363

Harbord: bias = 3,061897 (92,5% CI = -2,451848 to 8,575643) P = 0,302

*Trisomy 21*

Bias indicators

Begg-Mazumdar: Kendall's 0,296703 P = 0,1572

Egger: bias = 2,842155 (95% CI = -0,300921 to 5,98523) P = 0,0723

Harbord: bias = 2,350708 (92,5% CI = -3,168211 to 7,869627) P = 0,4226

*Trisomy 18*

Bias indicators

Begg-Mazumdar: Kendall's 0,186813 P = 0,388

Egger: bias = 1,162834 (95% CI = 0,234947 to 2,090722) P = 0,0182

Harbord: bias = 1,516873 (92,5% CI = -0,542002 to 3,575747) P = 0,1765

*Trisomy 13*

Bias indicators

Begg-Mazumdar: Kendall's 0,340659 P = 0,101

Egger: bias = 0,742524 (95% CI = -0,130313 to 1,61536) P = 0,0885

Harbord: bias = 1,229927 (92,5% CI = -1,202489 to 3,662342) P = 0,3437

*Sex chromosomal anomalies*

Bias indicators

Begg-Mazumdar: Kendall's 0,028571 P = 0,9226

Egger: bias = 0,495464 (95% CI = 0,000896 to 0,990032) P = 0,0496

Harbord: bias = 0,60849 (92,5% CI = -0,371738 to 1,588718) P = 0,251

*Rare autosomal anomalies*

Begg-Mazumdar: Kendall's 0,333333 P = 0,1289

Egger: bias = 0,415332 (95% CI = -0,059228 to 0,889893) P = 0,0803

Harbord: bias = 0,526506 (92,5% CI = -1,383446 to 2,436459) P = 0,5986

*Pathogenic or likely pathogenic copy number variants*

Bias indicators

Begg-Mazumdar: Kendall's -0,2 P = 0,2816

Egger: bias = 2,298474 (95% CI = 1,344862 to 3,252086) P = 0,0002

Harbord: bias = 2,88485 (92,5% CI = 0,049745 to 5,719955) P = 0,0706

***Al fetuses with NT between 3.0 and 3.4 mm (application of cfDNA)***

*All anomalies*

*Common tirsomies*
